# Noninvasive Relative Quantification of [^11^C]ABP688 PET Imaging in Mice Versus an Input Function Measured Over an Arteriovenous Shunt

**DOI:** 10.3389/fneur.2018.00516

**Published:** 2018-06-29

**Authors:** Jeroen Verhaeghe, Daniele Bertoglio, Lauren Kosten, David Thomae, Marleen Verhoye, Annemie Van Der Linden, Leonie Wyffels, Sigrid Stroobants, John Wityak, Celia Dominguez, Ladislav Mrzljak, Steven Staelens

**Affiliations:** ^1^Molecular Imaging Center Antwerp, University of Antwerp, Wilrijk, Belgium; ^2^Department of Nuclear Medicine, Antwerp University Hospital, Edegem, Belgium; ^3^Bio-Imaging Lab, University of Antwerp, Wilrijk, Belgium; ^4^CHDI Foundation, Princeton, NJ, United States

**Keywords:** [^11^C]ABP688, input function, mGluR5, PET imaging, test-retest

## Abstract

Impairment of the metabotropic glutamate receptor 5 (mGluR5) has been implicated with various neurologic disorders. Although mGluR5 density can be quantified with the PET radiotracer [^11^C]ABP688, the methods for reproducible quantification of [^11^C]ABP688 PET imaging in mice have not been thoroughly investigated yet. Thus, this study aimed to assess and validate cerebellum as reference region for simplified reference tissue model (SRTM), investigate the feasibility of a noninvasive cardiac image-derived input function (IDIF) for relative quantification, to validate the use of a PET template instead of an MRI template for spatial normalization, and to determine the reproducibility and within-subject variability of [^11^C]ABP688 PET imaging in mice. Blocking with the mGluR5 antagonist MPEP resulted in a reduction of [^11^C]ABP688 binding of 41% in striatum (*p* < 0.0001), while no significant effect could be found in cerebellum (−4.8%, *p* > 0.99) indicating cerebellum as suitable reference region for mice. DVR-1 calculated using a noninvasive IDIF and an arteriovenous input function correlated significantly when considering the cerebellum as the reference region (striatum: DVR-1, *r* = 0.978, *p* < 0.0001). Additionally, strong correlations between binding potential calculated from SRTM (BP_ND_) with DVR-1 based on IDIF (striatum: *r* = 0.980, *p* < 0.0001) and AV shunt (striatum: *r* = 0.987, *p* < 0.0001). BP_ND_ displayed higher discrimination power than V_T_ values in determining differences between wild-types and heterozygous Q175 mice, an animal model of Huntington's disease. Furthermore, we showed high agreement between PET- and MRI-based spatial normalization approaches (striatum: *r* = 0.989, *p* < 0.0001). Finally, both spatial normalization approaches did not reveal any significant bias between test-retest scans, with a relative difference below 5%. This study indicates that noninvasive quantification of [^11^C]ABP688 PET imaging is reproducible and cerebellum can be used as reference region in mice.

## Introduction

Glutamate is the most prominent neurotransmitter in the brain. The metabotropic glutamate receptors (mGluRs) are G-protein coupled receptors which modulate synaptic transmission and neuronal excitability (1). Impairment of the mGluR Group I (mGluR1 and mGluR5) has been implicated with various neurologic disorders, including Huntington's disease (HD) (2). HD is an autosomal dominant neurodegenerative disorder (3) caused by an expanded CAG repeat in exon 1 of the gene encoding the protein huntingtin (HTT) (4). Subjects with HD exhibit as main neuropathological feature a progressive neuronal cell loss in the caudate-putamen (5), which receives input from different areas of the basal ganglia as well as glutamatergic inputs from thalamus and cortex (6). Thus, mGluR5 is hypothesized to play an important role in the pathogenesis of HD (7, 2) and it represents an interesting target to image *in vivo* by means of positron emission tomography (PET). Among the developed radiotracers to image mGluR5, there is [^11^C]ABP688 (3-(6-methyl-pyridin-2-ylethynyl)-cyclohex-2-enone-O-^11^C-methyl-oxime), which binds to the mGluR5 allosteric-binding site with high affinity and selectivity (8). Although [^11^C]ABP688 PET imaging has successfully been used to quantify mGluR5 at preclinical and clinical level (8–11), the methods for reproducible quantification of [^11^C]ABP688 PET imaging in mice have not been thoroughly investigated yet.

Volume of distribution (V_T_) quantification requires the knowledge of the arterial input function (IPF), which can be measured by serial arterial blood sampling. However, in small animals, the amount of blood is very limited and therefore we applied an arteriovenous (AV) shunt coupled with a γ coincidence counter (12). The AV shunt surgery in small animals is typically an end of life procedures and does not allow longitudinal imaging over months in the same animals. A noninvasive approach is the extraction of the whole blood input function from the PET images. This image-derived input function (IDIF) is typically obtained from the lumen of the left ventricle of the heart (13) end-of-diastole in ECG monitored animals. In order to assess [^11^C]ABP688 quantification for longitudinal studies, first we determined the reproducibility of the IDIF by comparison with blood sampling via AV shunt and γ coincidence counter.

Nevertheless, the need of an IPF could be overcome altogether in the presence of a reference region in the brain devoid of the targeted receptors by using reference tissue models (14). Previous studies with [^11^C]ABP688 have shown cerebellum or cerebellar gray matter to be an optimal reference region in rats (10), baboons (11), and in humans (15) although mGluR5 is known to be present in small quantities in the cerebellum (16, 17). The valid use of a reference region for [^11^C]ABP688 quantification has not been investigated in mice. For this reason, the second aim of this study was to evaluate and validate the reference tissue model with the cerebellum as reference region for [^11^C]ABP688 PET imaging in mice.

Additionally, as HD is characterized by brain atrophy, primarily in striatum and cortex (5), we investigated the potential benefit of individual magnetic resonance (MR) images compared to the use of a [^11^C]ABP688 PET template for spatial normalization for quantification of [^11^C]ABP688 PET binding in the mouse brain.

Previous preclinical test-retest studies in rats and baboons indicated that [^11^C]ABP688 provides reproducible outcome measures with an average percentage difference below 10% (11, 18). On the other hand, same day test-retest studies in rhesus monkeys and in humans reported larger intra-individual variability, with an average increased in [^11^C]ABP688 uptake during the retest scan (15, 19, 20). As test-retest stability of [^11^C]ABP688 has never been investigated in mice, the final aim of this study was to determine the reproducibility and within-subject variability.

## Materials and methods

### Animals

Six months old (*n* = 29 per genotype) and 9 months old (*n* = 4 per genotype) male heterozygous (HET) Q175 mice (21, 22) and wild-type (WT) Q175 littermates were obtained from Jackson Laboratories (Bar Harbour, Maine, USA) were included in this study. Animals were single-housed in individually ventilated cages under a 12 h light/dark cycle in a temperature- and humidity-controlled environment with food and water *ad libitum*. The animals were acclimatized to the facility for at least 1 week before the start of procedures, which were performed according to the European Committee Guidelines (decree 2010/63/CEE) and the Animal Welfare Act (7 USC 2131). All experiments were in compliance with the ARRIVE guidelines, and they were approved by the Ethical Committee for Animal Testing (ECD 2014-92) at the University of Antwerp (Belgium) and all applicable institutional and European guidelines for the care and use of animals were followed.

### Study design

To assess noninvasive quantification of [^11^C]ABP688, HET and WT Q175 mice (*n* = 6 per genotype, 6 months old) were scanned with an AV shunt in order to determine V_T_ invasively and compare it to noninvasive approaches, namely V_T_ with IDIF and non-displaceable binding potential (BP_ND_) calculated from distribution volume ratio-1 (DVR-1) as well as using the simplified reference tissue model (SRTM). Comparison of discrimination power between genotypes was performed for the different quantification methods. To validate cerebellum as reference region, a blocking experiment with an mGluR5 antagonist was performed and compared to baseline scans (*n* = 4 per condition, 9 months old). In order to determine the accuracy of a [^11^C]ABP688 PET template for quantifying mGluR5 the V${}_{{\text{T}}^{\text{IDIF}}}$ in different regions was quantified in a total of 36 mice (HET and WT Q175, *n* = 18 per genotype, 6 months old) following two different spatial normalization approaches based respectively on individual MR images or the aforementioned [^11^C]ABP688 PET template. Finally, for test-retest stability of [^11^C]ABP688, a total of 10 mice were included (HET and WT Q715, *n* = 5 for each genotype, 6 months old).

### Tracer radiosynthesis

[^11^C]ABP688 was prepared using an automated synthesis module (Carbosynthon I, Comecer, The Netherlands). Synthesis of [^11^C]ABP688 was accomplished by reacting of 0.5 mg desmethyl-ABP-688 (E/Z) with [^11^C]CH_3_SO_3_CF_3_ in 400 μl of acetone in presence of 10 μl of NaOH, followed by purification and filtration as previously described (8). Average radiochemical purity was 98.6 ± 1.2%, while the mean specific radioactivity was 73 ± 16 GBq/μmol.

### [^11^C]ABP688 dynamic microPET scan

MicroPET/Computed tomography (CT) imaging was performed on two Siemens Inveon PET-CT scanners (Siemens Preclinical Solution, USA). The animals were anesthetized using isoflurane (Forene, Belgium) in medical oxygen (induction 5%, maintenance 1.5%), catheterized in the tail vein for intravenous (i.v) bolus injection of the tracer and positioned onto the scanner. Respiration and heart rate of the animal were constantly monitored using the Monitoring Acquisition Module (Minerve, France), with body temperature of the animals maintained at 37 ± 1°C using a feedback-controlled warm air flow (Minerve, France) during the entire scanning period. A full body image was acquired in a single PET bed position, thus including the lumen of the left ventricle of the heart in the field of view (FOV) for the calculation of the IDIF.

To measure the arterial input function, an AV shunt was surgically inserted into the femoral vein and artery prior to PET scan. After positioning the animal onto the scanner, the shunt was connected to a peristaltic pump: tubing from the artery was led through the Twilite detector (23) and ran through the pump. The tubing coming from the vein was connected on the output line of the pump, together with a second line for tracer injection with a bolus of [^11^C]ABP688. During injection, the peristaltic pump was stopped to prevent backflow. To perform [^11^C]ABP688 dynamic microPET scan with cardiac gating, cardiac electrocardiogram (ECG) signal were obtained from electrode tubes covered with ECG gel that were placed over the animal's front legs and one hind leg.

At the onset of the 60 min dynamic microPET scan, mice were injected with a bolus of [^11^C]ABP688 over a 12 s interval (1 ml/min) using an automated pump (Pump 11 Elite, Harvard Apparatus, USA). Tracer activity was injected keeping the cold dose within tracer conditions (< 1.50 μg/kg). PET data were acquired in list mode and the cardiac gate trigger signals were inserted into the list mode stream. Following the microPET scan, a 10 min 80 kV/500 μA CT scan was performed for attenuation and scatter correction. The AV shunt surgery was performed in the animals allocated for the first study (invasive quantification of [^11^C]ABP688), while the cardiac gating was included in the test-retest study. For the blocking experiment, the highly selective non-competitive mGluR5 antagonist 1,2-methyl-6-(phenylethynyl)-pyridine (MPEP) was used. MPEP was dissolved in saline and administered with an i.v. bolus injection (6 mg/kg) 10 minutes before the injection of the radiotracer. To limit the possible effect of a circadian variation in mGluR5 availability, test-retest scans were acquired at similar time of the day, while the scans for the blocking experiment were performed at the exact same time of the day.

A total of 3 WT and 2 HET Q175 mice were excluded from the [^11^C]ABP688 PET template validation study due to issues related to either tracer injection or image acquisition. Data on the body weight of the animals, injected radioactivity, injected mass, and number of animals for each study are reported in Supplementary Table 1.

### Image processing and analysis

Acquired PET data were histogrammed and reconstructed into 33 frames of increasing duration (12 × 10 s, 3 × 20 s, 3 × 30 s, 3 × 60 s, 3 × 150 s, and 9 × 300 s). Iterative PET image reconstruction of the images was performed using 4 iterations and 16 subsets of the 2-dimensional ordered-subset expectation maximization (2D-OSEM) algorithm (24) following Fourier rebinning. Normalization, dead time, CT-based attenuation and single-scatter simulation scatter corrections were applied. PET image frames were reconstructed on a 128 × 128 × 159 grid with 0.776 × 0.776 × 0.776 mm^3^ ignoring the cardiac gating trigger signals. These reconstructions were used for quantifying the brain uptake. Additional cardiac gated reconstruction was also obtained by dividing each heart cycle (defined by the gating trigger signals in the list-mode stream) into 4 bins and the bin containing the diastole images were used to derive the IDIF.

The arterial IPF was obtained at a 1 s sampling interval from the whole blood activity derived from the Twilite count detection coupled with the AV shunt. To reduce the noise in the Twilite data, a three-exponential function was fitted to the decaying part of the IPF. The delay between the IPF measured at the shunt and the true cerebral IPF was estimated trough a two-tissue compartmental model fit with an extra free parameter for the delay to the striatal and cortical time activity curves (TACs). The same delay was shared between the different regions. The IDIF was obtained from the whole blood activity derived from the PET images by delineating a region-of-interest (threshold set to 50% of max) in the lumen of the left ventricle of the heart. The ventricular region was delineated on an early time frame exhibiting maximal activity in the lumen of the left ventricle.

PET images were processed and analyzed using PMOD 3.6 software (Pmod Technologies, Zurich, Switzerland). For spatial normalization of the PET/CT images, a [^11^C]ABP688 PET template was generated using only the data of the WT animals (*n* = 16). First, individual static PET images covering the whole scan duration (i.e., 60 min) were generated and spatially aligned to their individual MRI by applying the non-linear warping CT to MRI transformation. The transformation was calculated by co-registering the individual animals CT image to its corresponding MR image. The same transformation could be used to align the PET images as the PET/CT images were intrinsically co-registered as acquired on the same gantry. Then, all individual MR images were spatially transformed through a rigid body registration to the MRI of the first animal. The MR to MR transformations obtained during creation of the WT MR template were then also applied to the corresponding static PET images. The static PET MR images (*n* = 16) were averaged resulting in a [^11^C]ABP688 WT template ([^11^C]ABP688 PET template). To validate the [^11^C]ABP688 PET template, spatial normalization of the PET/CT images was performed through rigid body image co-registration of the PET images to both (i) individual MR images (based on the CT to MR transformation) and (ii) [^11^C]ABP688 PET template (based on a transformation calculated from PET to PET image registration). Following validation, the [^11^C]ABP688 PET template was applied to all other PET images for quantification (i.e., test-retest study, validation IDIF, and DVR-1 vs. SRTM comparison). The volumes-of-interest (VOIs) were determined based on an existing MRI template with predefined VOIs (25). Using the predefined VOIs of the template, standardized uptake value (SUV) TACs of different regions (striatum, cerebral cortex, hippocampus, thalamus, and cerebellum) were extracted from the images. Following kinetic modeling, TACs were fitted by a standard two-tissue compartment model (2TCM) with blood volume fixed at 0.036 mL/cm^3^ and by a Logan model (26) to calculate the total volume of distribution V_T_ using the measured arterial IPF and IDIF (V${}_{{\text{T}}^{\text{inv}}}$ and V${}_{{\text{T}}^{\text{IDIF}}}$). As there was a high correlation between 2TCM and Logan (e.g., in striatum: *r* = 0.998, *r*^2^ = 0.997), the regression line was close to identity line, and the Bland-Altman plot showing limited bias (4.09%) as well as negligible 95% confidence intervals (from 1.81 to 6.38%), only V_T_ calculated with Logan is reported. From the V_T_ values, DVR-1 was calculated with the cerebellum as reference region. In addition, the binding potential BP_ND_ for these regions was calculated using the SRTM (14) with the cerebellum as reference tissue.

V_T_, DVR-1 and BP_ND_ values were calculated to validate IDIF as input function for kinetic modeling. V_T_ values were calculated for the blocking experiment to validate the presence of reference region. V_T_ values obtained from either the individual MRI or PET template approach, and they were compared to evaluate the accuracy of the [^11^C]ABP688 PET template for spatial normalization. Finally, V_T_ and BP_ND_ values were calculated to determine test-retest stability of [^11^C]ABP688 PET imaging.

Additionally, voxel-based parametric V_T_ images were generated using Logan model with the IDIF as input function, while BP_ND_ images were calculated using the SRTM with the cerebellum as reference region. Parametric images are represented as averages over the group (HET and WT) in stack coronal slices selected from a 3D coronal/sagittal/transversal mouse brain view.

### T_2_-weighted MRI

To validate the [^11^C]ABP688 template for PET quantification, individual MR images were obtained in the same week of the microPET/CT scan. The animals were anesthetized using isoflurane in a mixture of N_2_/O_2_ (induction 5%, maintenance 1.5%) and placed in prone position onto the scanner (7T Biospec, Bruker, Germany). Body temperature was maintained at 37 ± 1°C by means of rectal thermistor with a feedback-controlled warm air circuitry (MR-compatible Small Animal Heating System, SA Instruments, Inc. USA). Three-dimensional turbo rapid acquisition with relaxation enhancement (turboRARE) images were acquired with repetition time 3,185 ms, echo time 44 ms, echo train length 8, and matrix size 128 × 64 × 40. FOV was 25.6 × 13 × 10 mm^3^ and resolution of 0.2 × 0.2 × 0.25 mm^3^. The MR image acquisition procedure lasted 21 min. Data were acquired using ParaVision 5.1 (Bruker, Germany).

### Statistical analysis

All data were assessed for normality (Shapiro-Wilk test). Since no evidence against normality was found, parametric tests were performed. Differences between V_T_ quantification based on either AV shunt or IDIF were evaluated separately with the paired-*t* test for each region. Two-way ANOVA was applied to the blocking experiment to compare baseline and blockade scans in the different regions. Pearson's correlation tests were used to determine the relationship between V_T_ and DVR-1 quantified with the different input functions (namely, AV shunt and IDIF), between DVR-1 and BP_ND_, and to determine correlations between V_T_ values obtained with both the individual MRI and PET template. Additionally, the agreement between individual V_T_ measurements based on MRI and PET templates was visualized by plotting the percentage difference between the two parameters against their averages in a Bland-Altman plot (27). For the test-retest study, reproducibility of the data was determined by the intraclass correlation coefficient (ICC). Pearson's correlation tests as well as Bland-Altman plots were used to compare V_T_ and BP_ND_ values for test-retest scans. In addition, the percentage relative change between test and retest were calculated as relative difference = |retest – test|/retest x 100%. Finally, the mean ± standard deviation (SD) of the intra-animal coefficient of variation (COV) was calculated as follow:

(1) $$\begin{array}{r} COV_{G}=\frac{1}{N}\overset{N}{\underset{i}{\sum }}\frac{SD_{i}^{G}}{{\bar{x}}_{i}^{G}} \end{array}$$

where *G* represents the group, *N* is the number of animals in the group, ${\bar{x}}_{i}^{G}$and $SD_{i}^{G}$ are respectively the mean and standard deviation of the test and retest values for animal *i*. Paired *t*-test was performed to investigate any methodological difference between test and retest scans. All analyses were performed with GraphPad Prism (v 6.0) statistical software, with the exception of the ICC, which were calculated in JMP Pro 13 software (SAS Institute Inc., USA) and the power analysis, which was calculated with G^*^Power software (http://www.gpower.hhu.de/). The data are represented as mean ± standard deviation (SD), unless specified otherwise. All tests were two-tailed and significance was set at *p* < 0.05.

## Results

### Quantification based on IDIF and IPF correlate significantly when using cerebellum as the reference region

**Figure 1:**
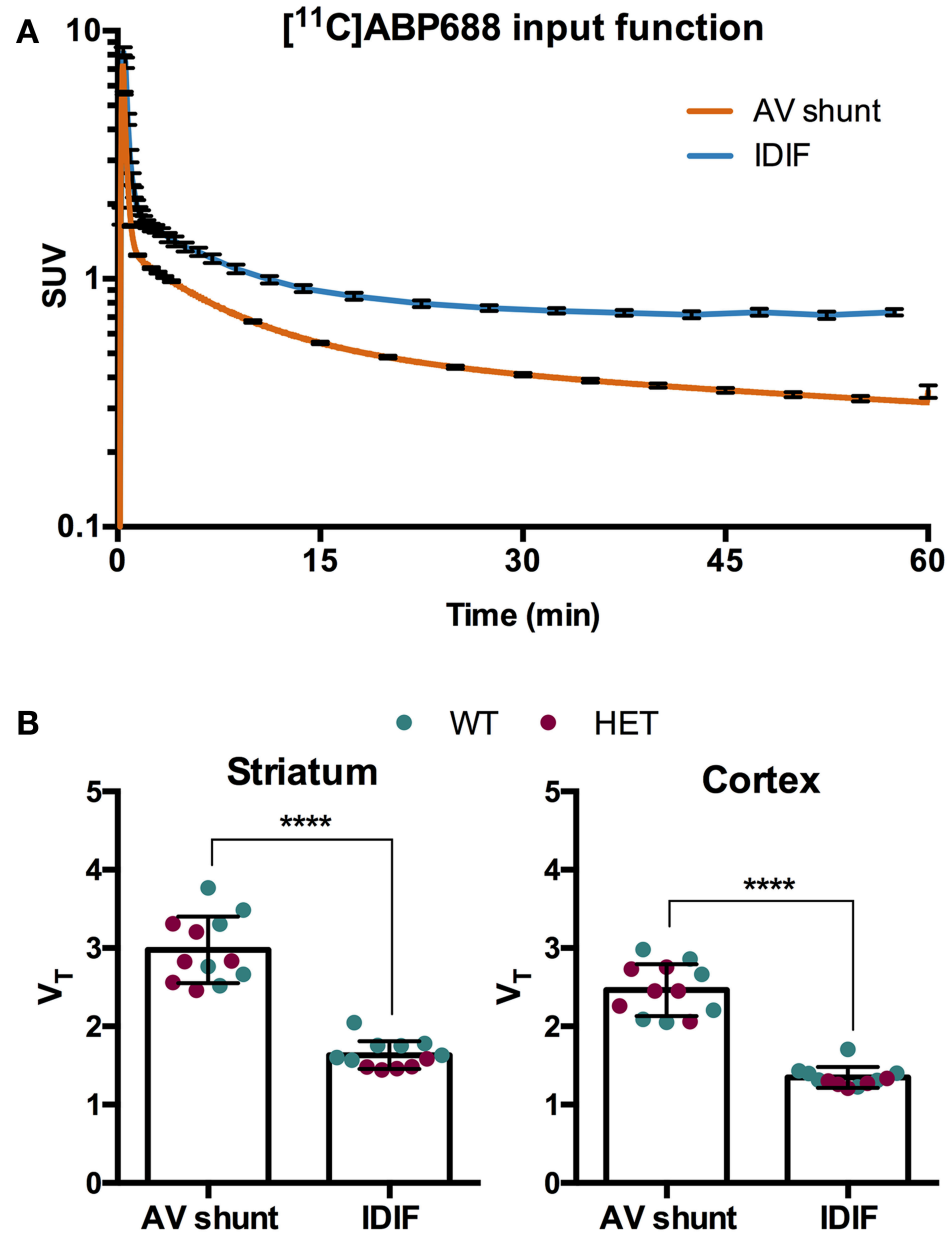
Comparison of invasive and noninvasive input functions for [^11^C]ABP688 quantification. **(A)** Average SUV TACs of the IPF based on arteriovenous (AV) shunt and image-derived input function (IDIF) (*n* = 12) (note the logarithmic scale on the y-axis). **(B)** Comparison of volume of distribution (V_T_) quantification of [^11^C]ABP688 based on Logan plot in striatum (left) and cortex (right). As a consequence of the overestimation of the tail of the IPF by the IDIF, the V_T_ is significantly lower compared to V_T_ based on AV shunt IPF. Paired-*t* test (*n* = 12). ****p < 0.0001. IPF, input function; WT, wild type; HET, heterozygous.

**Figure 2:**
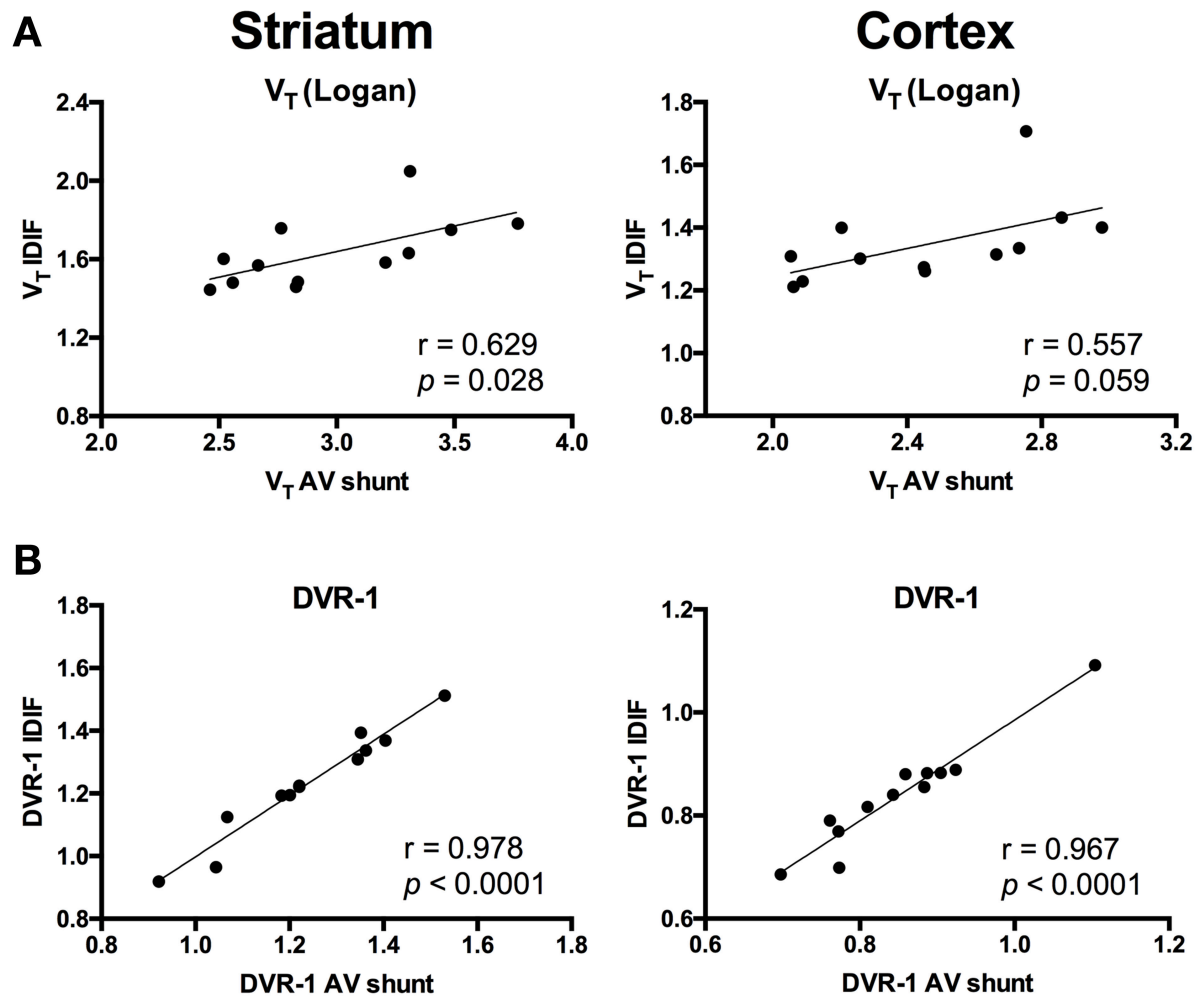
Correlations between invasive and noninvasive [^11^C]ABP688 quantification. **(A)** Volume of distribution (V_T_) values showed a moderate correlation when comparing the 2 input function approaches in both striatum (left) and cortex (right). **(B)** Distribution volume ratio (DVR-1) of [^11^C]ABP688 displayed strong correlations between input function based on arteriovenous shunt and image-derived input function in both striatum (left) and cortex (right). Pearson's correlation test.

To evaluate a noninvasive approach for quantification of [^11^C]ABP688, arterial IPF based on AV shunt and IDIF were compared. Average input function TACs for AV shunt and IDIF approaches are shown in Figure 1A. The IDIF tail values were higher than the AV shunt values at corresponding time points, indicating that IDIF overestimates the radioactivity present in the blood (Figure 1A). Accordingly, V_T_ values using IDIF are significantly lower (*p* < 0.0001) than with an AV shunt IPF in both WT and HET Q175 mice (*n* = 6 per genotype; Figure 1B). As shown in Figure 2A and reported in Supplementary Table 2, moderate correlations were found between V_T_ values obtained from the 2 input functions in striatum (*r* = 0.629, *p* = 0.028), cortex (*r* = 0.557, *p* = 0.059), hippocampus (*r* = 0.558, *p* = 0.059), and thalamus (*r* = 0.536, *p* = 0.072). However, when considering the cerebellum as reference region, correlations between DVR-1 based on AV shunt and IDIF were strong and highly significant in all regions (*r* = 0.978, *p* < 0.0001 in striatum; *r* = 0.967, *p* < 0.0001 in cortex; *r* = 0.955, *p* < 0.0001 in hippocampus; *r* = 0.934, *p* < 0.0001 in thalamus) (Figure 2B and Supplementary Table 2).

**Figure 3:**
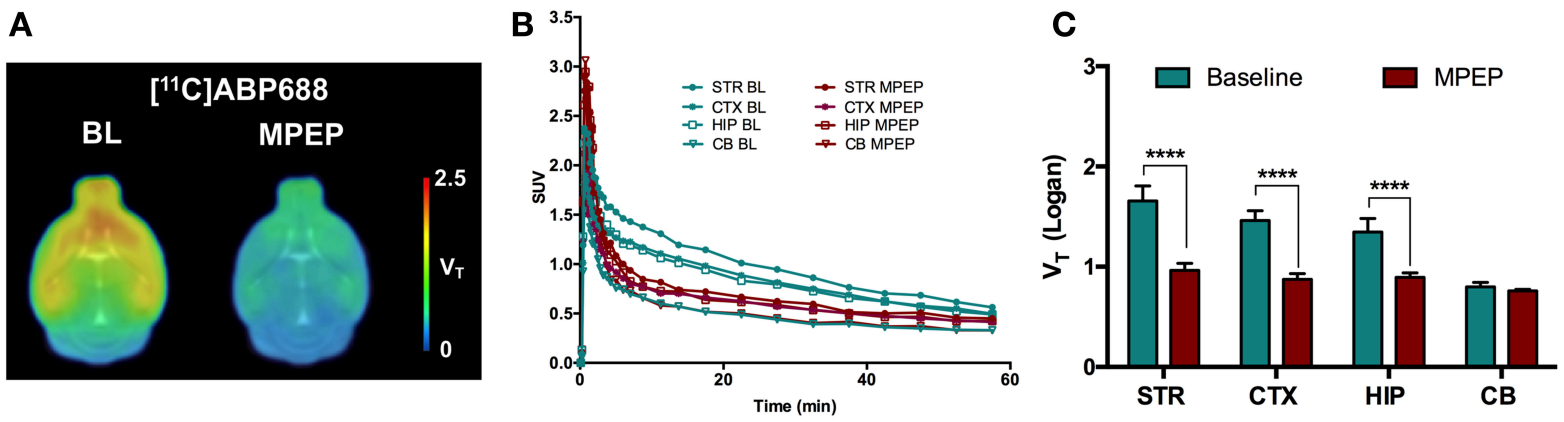
Validation of cerebellum as reference region for [^11^C]ABP688 in mice. **(A)** Average parametric V_T_ images during baseline scan (BL) and following administration of MPEP (6 mg/kg) 10 min before tracer injection in WT mice. **(B)** SUV time-activity curves for different brain region during baseline and blocking scans. **(C)** V_T_ (Logan) quantification showed a statistically significant reduction in mGluR5-rich regions, while no significant changes were found in cerebellum. *n* = 4 per condition. ****p < 0.0001. BL, baseline; MPEP, 1,2-methyl-6-(phenylethynyl)-pyridine; STR, striatum; CTX, cortex; HIP, hippocampus; CB, cerebellum.

A blocking experiment was performed to investigate cerebellum as a possible reference region in mice (Figure 3). Average standardized uptake value (SUV) time-activity curves of animals injected with MPEP displayed a clear reduction of [^11^C]ABP688 binding compared to baseline in striatum, cortex, and hippocampus, while in cerebellum no reduction could be observed (Figure 3B). Accordingly, MPEP administration resulted in a statistically significant decrease of [^11^C]ABP688 binding in receptor-rich regions (striatum: −42%, *p* < 0.0001; cortex: −40%, *p* < 0.0001; hippocampus: −33%, *p* < 0.0001). On the contrary, in the cerebellum only a negligible no significant reduction of V${}_{\text{T}}^{\text{IDIF}}$ (Logan) could be observed (−4.8%, *p* > 0.99) (Figure 3C).

**Figure 4:**
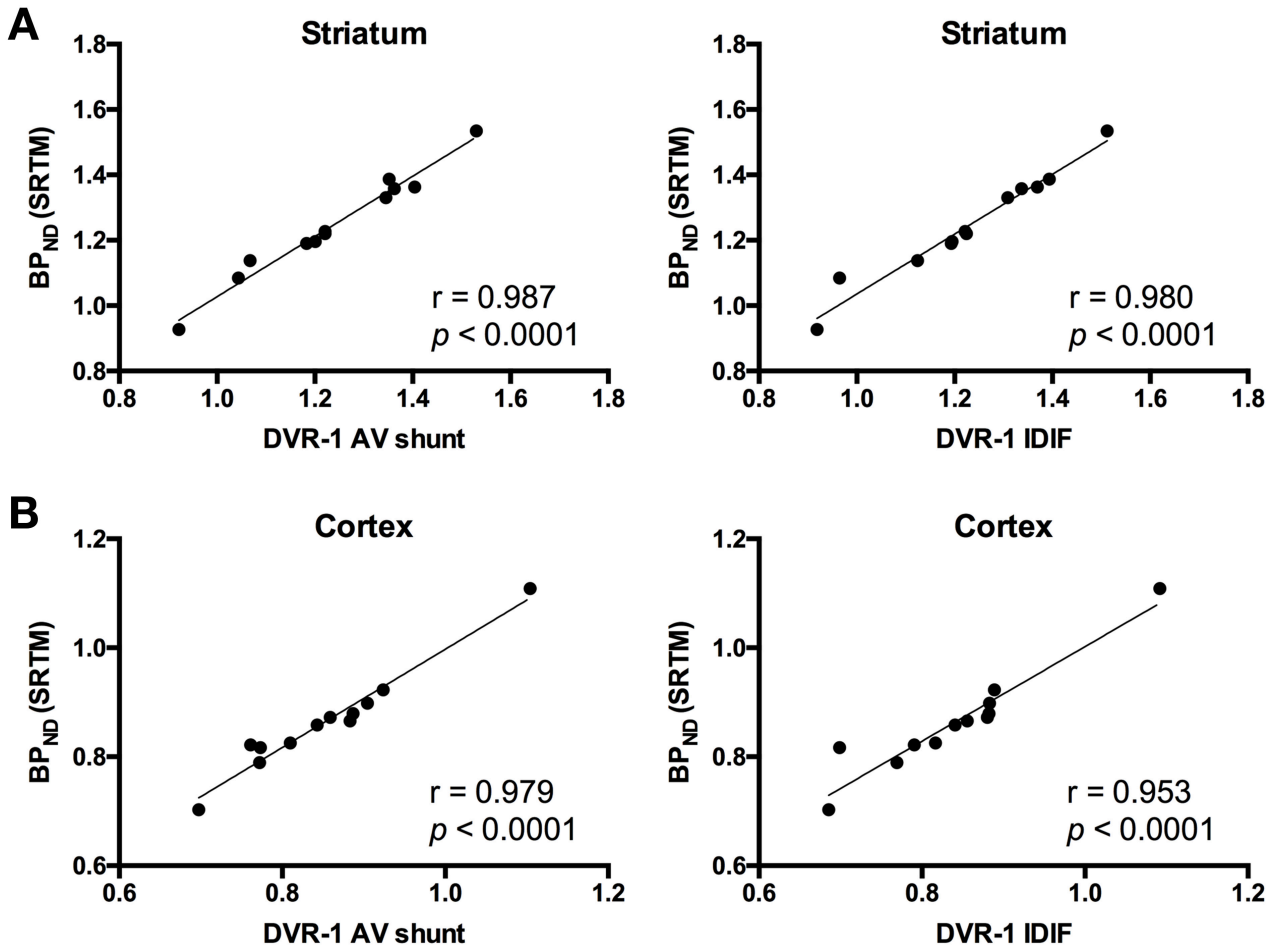
Correlations between distribution volume ratio (DVR-1) and binding potential (BP_ND_) values for [^11^C]ABP688. In striatum **(A)** and cortex **(B)**, DVR-1 calculated with AV shunt as well as IDIF strongly correlated with BP_ND_ determined using the simplified reference tissue model (SRTM). Pearson's correlation test. AV, arteriovenous; IDIF, image-derived input function.

As the administration of mGluR5 antagonist MPEP did not modify V${}_{{\text{T}}^{\text{IDIF}}}$ values in cerebellum, we selected this region as reference region for relative quantification. Thus, we quantified BP_ND_ with SRTM [BP_ND(SRTM)_] and compared it to the aforementioned DVR-1 values based on AV shunt and IDIF. Correlations between BP_ND(SRTM)_ and DVR-1 based on AV shunt were strong and highly significant in all the investigated regions (*r* = 0.987, *p* < 0.0001 in striatum; *r* = 0.979, *p* < 0.0001 in cortex; *r* = 0.976, *p* < 0.0001 in hippocampus; *r* = 0.953, *p* < 0.0001 in thalamus) (Figure 4 and Supplementary Table 3). Similarly, highly significant correlations were found between BP_ND(SRTM)_ and DVR-1 based on IDIF in all the investigated regions (*r* = 0.980, *p* < 0.0001 in striatum; *r* = 0.953, *p* < 0.0001 in cortex; *r* = 0.959, *p* < 0.0001 in hippocampus; *r* = 0.953, *p* < 0.0001 in thalamus) (Figure 4 and Supplementary Table 3).

Since both V${}_{{\text{T}}^{\text{inv}}}$, V${}_{{\text{T}}^{\text{IDIF}}}$, and BP_ND(SRTM)_ were obtained from the same animals, we investigated whether the use of an IDIF or BP_ND_ would affect the quantification of disease-related changes in WT and HET Q175 mice. As shown in Supplementary Figure 1, the genotypic difference in striatal V${}_{{\text{T}}^{\text{inv}}}$ and V${}_{{\text{T}}^{\text{IDIF}}}$ displayed was comparable (−7.0%, *p* = 0.40 and −5.9%, *p* = 0.35, respectively), while striatal BP_ND(SRTM)_ could detect a larger difference between genotypes (−10.3%, *p* = 0.16), however none of the comparison reached statistical significance due to the limited sample size (*n* = 6 per genotype). In accordance, the power analysis performed on these values confirmed the higher discrimination power for BP_ND(SRTM)_ compared to V${}_{{\text{T}}^{\text{inv}}}$ and V${}_{{\text{T}}^{\text{IDIF}}}$ (Supplementary Table 4).

### MRI and PET template-based spatial normalization approaches showed high agreement in [^11^C]ABP688 quantification

**Figure 5:**
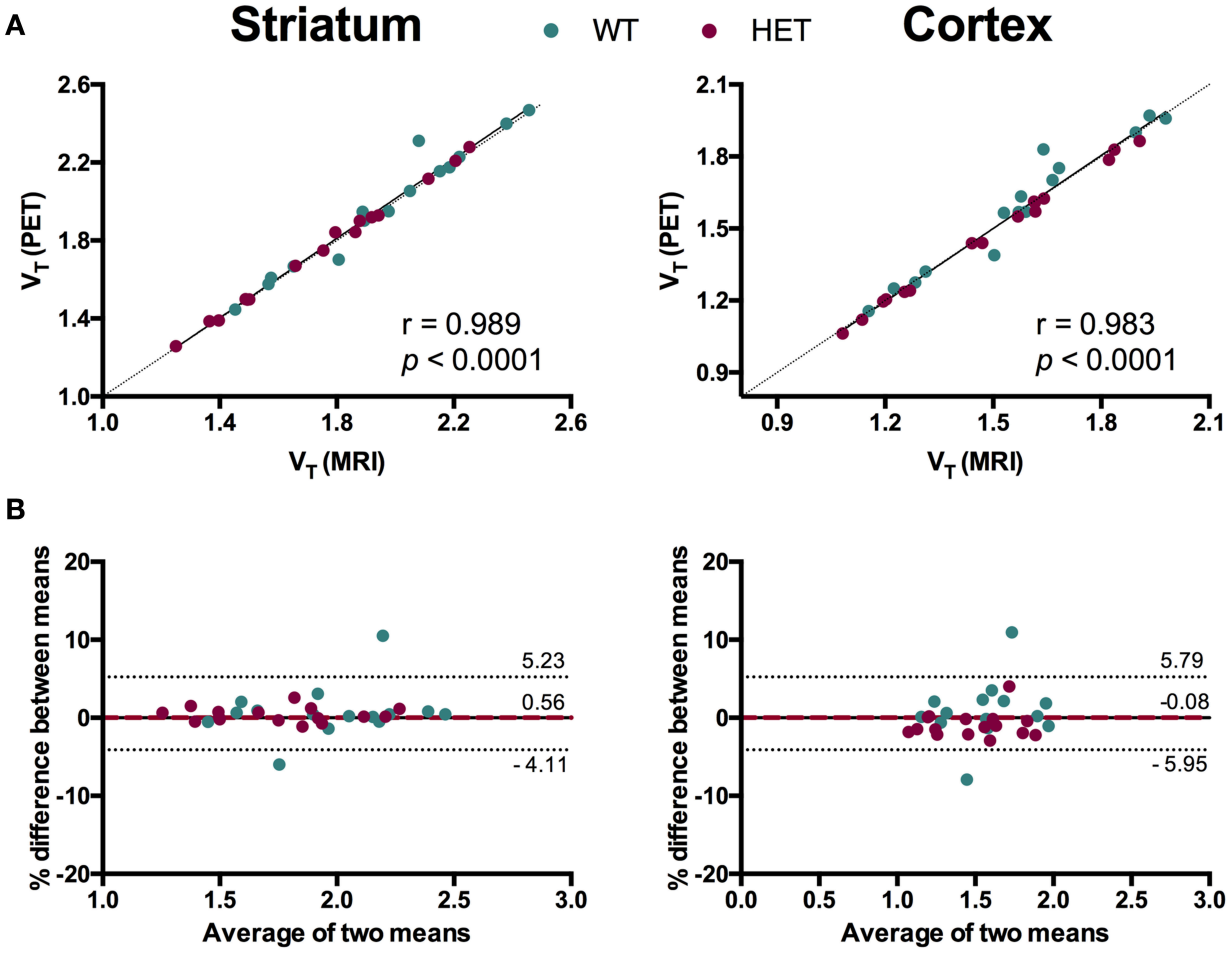
Comparison of spatial normalization approaches for [^11^C]ABP688 V_T_ regional quantification. **(A)** Correlation between V_T_ values in striatum (left) and cortex (right) based on individual MR images- and PET template-based spatial normalizations. Pearson's correlation test. Dashed line represents identity line. **(B)** Bland-Altman plot to compare the spatial normalization approaches in striatum (left) and cortex (right). The bias between the two approaches corresponds to the difference between the mean (red dashed line) and X axis (solid line). The dotted lines represent the 95% limits of agreement (mean difference ± 1.96 × SD of the differences), respectively. WT, wild type; HET, heterozygous.

To evaluate the necessity of individual MR images for [^11^C]ABP688 quantification, we compared V_T_ values based on individual MR images and the [^11^C]ABP688 PET template. The results of the different spatial normalization approaches for [^11^C]ABP688 are summarized in Figure 5 and Table 1. A strong and highly significant correlation was found between spatial normalization approaches in all investigated regions (*r* = 0.989, *p* < 0.0001 in striatum; *r* = 0.983, *p* < 0.0001 in cortex; *r* = 0.985, *p* < 0.0001 in hippocampus; *r* = 0.986, *p* < 0.0001 in thalamus) (Table 1). The correlations between approaches coincided with the identity line (Figure 5A). In addition, the Bland-Altman plot showed high agreement between the two approaches as visible by the low bias obtained (red dashed line; Figure 5B), which was ≤ 1% for all the investigated regions (Table 1).

**Table 1:** Relationship between MRI- and PET template-based spatial normalization for [^11^C]ABP688.

**Model**	**Region**	***r***	***r*^2^**	***p-*value**	**Bias (%)**	**LoA**
^V^T ^(Logan)^	Striatum	0.989	0.978	< 0.0001	0.56	−4.11; 5.23
	Cortex	0.983	0.967	< 0.0001	−0.08	−5.95; 5.79
	Hippocampus	0.985	0.970	< 0.0001	1.00	−4.57; 6.58
	Thalamus	0.986	0.973	< 0.0001	−0.87	−6.43; 4.67

*V_T_, volume of distribution; r, Pearson's correlation; r^2^, coefficient of determination; LoA, limits of agreement*.

### Noninvasive quantification of [^11^C]ABP688 imaging is reproducible

Methodologically no significant difference between test and retest scans was observed in the injected dose (5.24 ± 0.91 MBq and 4.89 ± 1.06 MBq, respectively; *p* = 0.39), specific activity (74.3 ± 18.5 GBq/μmol and 68.7 ± 14.2 GBq/μmol, respectively; *p* = 0.27), body weight (28.2 ± 1.8 g and 27.8 ± 1.6 g, respectively; *p* = 0.59) or injected mass (1.23 ± 0.08 μg/kg and 1.25 ± 0.05 μg/kg, respectively; *p* = 0.39). Finally, there was no significant difference between the start time for the test and retest scans (Δt = 59 ± 62 min; *p* = 0.62). Average TACs for striatum and cerebellum were consistent between test and retest scans and genotype as shown in Supplementary Figure 2.

**Figure 6:**
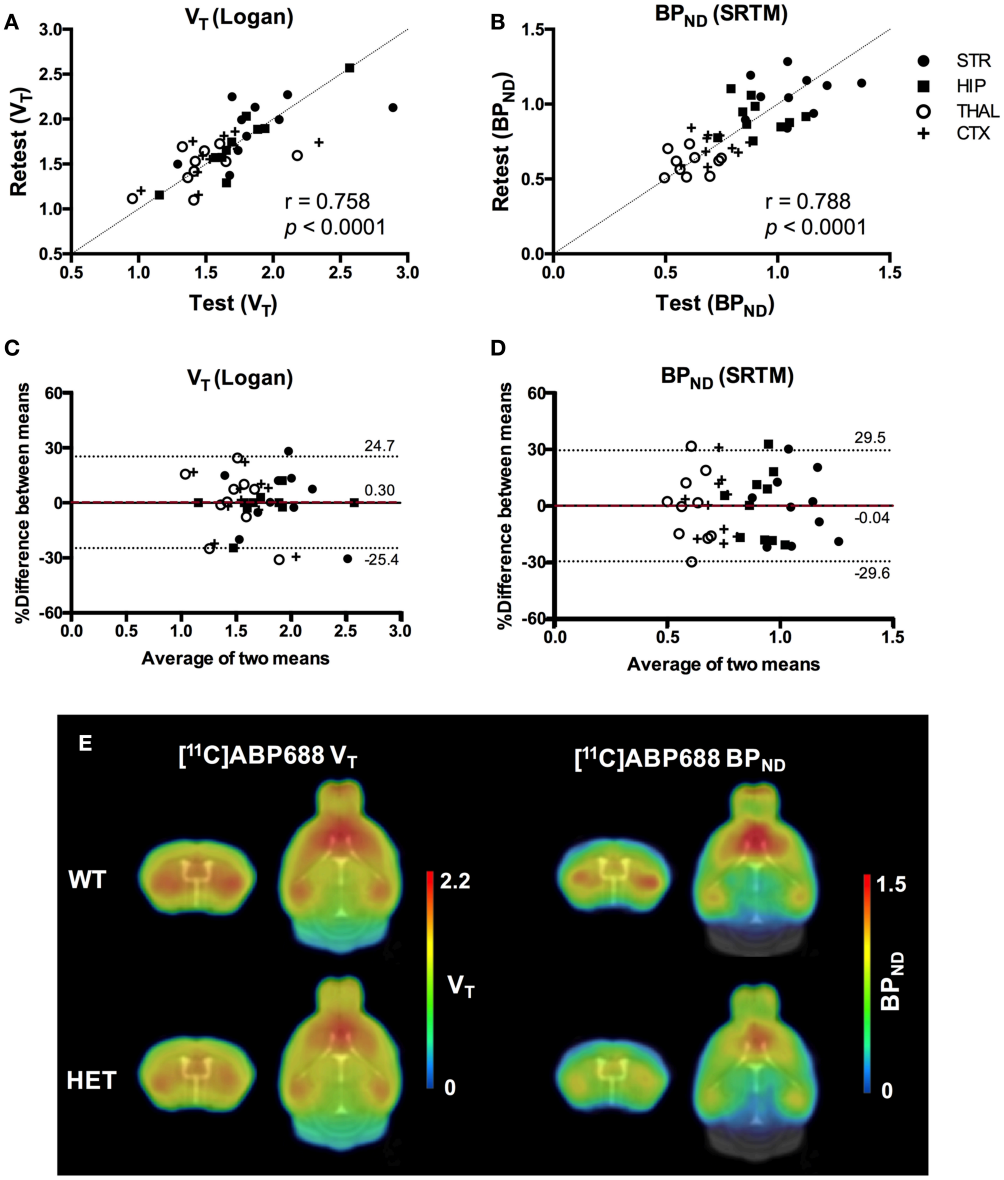
Reproducibility of test-retest quantification for [^11^C]ABP688. Correlations of the volume of distribution (V_T_) **(A)** and binding potential (BP_ND_) **(B)** for the regions of interest in both WT and HET Q175 mice (*n* = 5 per genotype). Pearson's correlation test. Bland-Altman plot to compare test-retest quantification of V_T_ **(C)** and BP_ND_ **(D)**. The bias between the two approaches corresponds to the difference between the mean (red dashed line) and X axis (solid line). The dotted lines represent the 95% limits of agreement (mean difference ± 1.96 x SD of the differences), respectively. **(E)** Average parametric images (*n* = 5) representing the V_T_ (left) and BP_ND_ (right) for both WT and HET Q175 mice. STR, striatum; HIP, hippocampus; THAL, thalamus; CTX, cortex; WT, wild type; HET, heterozygous.

Values and differences between test and retest scans are summarized in Table 2. For [^11^C]ABP688 V${}_{{\text{T}}^{\text{IDIF}}}$, the mean relative difference between test and retest was lower than 4.1% in all the investigated regions, with a SD ranging from 7.4 to 17.9%. ICC values were low in thalamus (0.35) and between 0.46 and 0.61 in the other regions (hippocampus, striatum and cortex, respectively). Intra-animal COV was lower than 10% in all investigated regions, with a SD ranging from 6.8 to 7.4%. In addition, the Bland-Altman plot showed a negligible bias between test and retest (0.30%), although the 95% confidence intervals were relatively large (−25.4%; 24.7%) (Figure 6).

**Table 2:** Reproducibility of test-retest parameters for [^11^C]ABP688 volume of distribution (V_T_) and binding potential (BP_ND_).

**Region**	**WT Q175**			**HET Q175**				
	**Test**	**Retest**	**Rel. Diff. (%)**	**Test**	**Retest**	**Rel. Diff. (%)**	**ICC**	**COV (%)**
	**Mean ± SD**	**Mean ± SD**		**Mean ± SD**	**Mean ± SD**			**Mean ± SD**
**V**_**T**_ **(LOGAN)**								
Striatum	1.95 ± 0.60	2.03 ± 0.32	3.83 ± 14.9%	1.83 ± 0.14	1.79 ± 0.30	1.92 ± 8.2%	0.57	9.4 ± 7.2%
Cortex	1.59 ± 0.49	1.63 ± 0.26	2.36 ± 15.1%	1.56 ± 0.13	1.52 ± 0.25	2.37 ± 8.3%	0.61	8.7 ± 6.8%
Hippocampus	1.76 ± 0.52	1.83 ± 0.28	3.91 ± 17.9%	1.74 ± 0.13	1.70 ± 0.28	1.99 ± 8.2%	0.46	9.3 ± 7.0%
Thalamus	1.49 ± 0.44	1.53 ± 0.24	2.28 ± 14.9%	1.46 ± 0.11	1.40 ± 0.20	4.07 ± 7.4%	0.35	9.1 ± 7.4%
**BP**_ND_ **(SRTM)**								
Striatum	1.16 ± 0.14	1.15 ± 0.09	1.17 ± 6.3%	0.97 ± 0.13	0.98 ± 0.14	1.09 ± 8.6%	0.54	9.9 ± 6.9%
Cortex	0.76 ± 0.08	0.74 ± 0.05	1.80 ± 5.7%	0.68 ± 0.10	0.69 ± 0.11	2.24 ± 9.6%	0.62	9.3 ± 6.1%
Hippocampus	0.96 ± 0.11	0.94 ± 0.14	2.48 ± 6.7%	0.85 ± 0.10	0.88 ± 0.14	3.49 ± 9.0%	0.53	9.7 ± 6.6%
Thalamus	0.65 ± 0.08	0.64 ± 0.06	2.67 ± 7.1%	0.57 ± 0.08	0.57 ± 0.08	0.59 ± 9.2%	0.32	10.2 ± 7.7%

*SD, standard deviation; WT, wild type; HET, heterozygous; Rel. Diff., relative difference; COV, coefficient of variation; ICC, intraclass correlation coefficient; V_T_, volume of distribution; BP_ND_, binding potential; SRTM, simplified reference tissue model*.

For [^11^C]ABP688 BP_ND(SRTM)_, the mean relative difference between test and retest was lower than 3.5% in all the investigated regions, with a SD ranging from 5.7 to 9.6%. ICC value was low in thalamus (0.32) and between 0.53 and 0.62 in the other regions (hippocampus, striatum and cortex, respectively). Intra-animal COV was lower than 10.2% in all investigated regions, with a SD ranging from 6.1 to 7.7%. Finally, the Bland-Altman plot confirmed the negligible bias between test and retest (−0.04%), despite the relatively large 95% confidence intervals (−29.6%; 29.5%) (Figure 6).

Scatter plots comparing the individual outcome values for both V_T_ and BP_ND_ and average parametric images for both V_T_ and BP_ND_ are shown in Figure 6.

## Discussion

The present study investigated several imaging parameters for reproducible quantification of [^11^C]ABP688 PET imaging in mice.

### Image-derived input function reproducibility and reference region validation for noninvasive quantification in mice

The employment of a noninvasive input function is extremely attractive for longitudinal PET imaging as it circumvents all challenges and limitations related to the arterial blood sampling. Thus, in the present study, we compared the invasive arterial IPF measured with AV shunt to a proposed noninvasive IDIF measured in the left ventricle of the heart. The IDIF resulted in a broader peak than the AV shunt. This can be attributed to the coarser time sampling and the contributions from both the left and right ventricular blood pools as well as the ventricular walls, three regions for which the peak activity concentration does not occur at the same time. The proposed IDIF resulted in an activity peak comparable to the AV shunt IPF, however, the IDIF overestimated the blood activity at the tails of the curve, resulting in higher values than the AV shunt IPF at corresponding times. This is likely due to spill over activity from nearby regions (e.g., myocardium) which is negligible at the time of the peak but becomes significant at later times. Since the difference between IDIF and AV shunt IPF increased during the scan, it was not possible to simply scale the IDIF e.g., using a single blood sample. As a consequence of the overestimation of the tail, V_T_ values calculated with IDIF were reduced compared to the values obtained with the AV shunt IPF (*p* < 0.0001). As a result, moderate correlations could be established between V_T_ values determined with IDIF and AV shunt (i.e., *r* = 0.629 in striatum). Nonetheless, when comparing the V_T_ values obtained from the 2 IPFs between WT and HET Q175 mice, where a reduction of mGluR5 binding is expected (28), the absolute genotypic V_T_ difference was comparable (e.g., in striatum, AV shunt: −7.0%, *p* = 0.40; IDIF: −5.9%, *p* = 0.36). This suggests that values determined with the IDIF, although they do not directly match those obtained using the AV shunt, might be representative of the quantification based on AV shunt IPF. However, future studies are necessary to investigate whether noninvasive IDIF could be employed as alternative to evaluate noninvasively group differences in longitudinal or interventional studies.

A limitation of the present study was the lack of metabolite-corrected IPF. This was not performed because of the limited amount of blood can be obtained from a specific mouse, which does not allow to collect multiple samples from the same animal when using standard techniques for metabolite analysis. One way to circumvent this limitation in future studies could be the use of different cohorts of animals in order to generate a population-based correction curve, possibly taking into account a potential age-dependent metabolism of the radiotracer.

Previous clinical and preclinical studies have reported the application of the cerebellum or cerebellar gray matter as reference region for [^11^C]ABP688 quantification (10, 15, 19). Therefore, we calculated DVR-1 values based on V_T_ from both IDIF and AV shunt using the cerebellum as reference region and we found strong and highly significant correlations between the 2 measurements (e.g., in striatum, *r* = 0.978; *p* < 0.0001). This finding indicates that DVR-1 quantification using IDIF provides highly comparable measurements to the arterial IPF and therefore it can be utilized for noninvasive quantification of [^11^C]ABP688 in the presence of a valid reference region. Nevertheless, the use of SRTM remains the preferable choice for noninvasive quantification of [^11^C]ABP688 if the reference region is validated. Validation of the reference region was performed with a blocking experiment by administration of the mGluR5 antagonist MPEP before the administration of the radiotracer. As the blocking of mGluR5 had a non-significant and negligible effect on cerebellar V_T_, we concluded that cerebellum can be used as reference region for [^11^C]ABP688 quantification. The 4.8% cerebellar reduction found in the present study is in line with the previously reported validation in rats where blocking with MPEP resulted in 7% reduction of the cerebellar V_T_ (based on Logan graphical analysis) (10). Other studies in baboons and humans validated the use of cerebellar gray matter as reference region (15, 11). Although the use of cerebellar gray matter might be more accurate, in mice a clear distinction between these regions is not possible due to the limited resolution of the PET camera. Thus, we validated the reference tissue model with the cerebellum as reference region by comparing BP_ND_ values calculated using the SRTM to the DVR-1 calculated with IDIF as well as with the AV shunt. As this resulted in strong and highly significant correlations (e.g., in striatum, *r* = 0.980 and *r* = 0.987, respectively). Interestingly, BP_ND(SRTM)_ resulted in a higher discrimination between genotypes (e.g., in striatum, −10.3%, *p* = 0.16) than the V_T_ quantification based on either AV shunt IPF or IDIF. Altogether these findings indicate that the SRTM using the cerebellum as reference region is suitable for quantification of [^11^C]ABP688 binding in mice.

### [^11^C]ABP688 PET template for spatial normalization

As a dedicated high resolution small animal MR scanner it is not always available to conduct preclinical PET studies and also in order to limit as much as possible the anesthesia sessions, we investigated the relevance of individual MRI-based spatial normalization to quantify [^11^C]ABP688. When comparing the MRI-based spatial normalization to the [^11^C]ABP688 PET template approach, we found that [^11^C]ABP688 V_T_ values strongly correlated (e.g., in striatum, *r* = 0.989, *p* < 0.0001), with no statistical significant difference in the quantification of [^11^C]ABP688. This finding indicates that PET-based spatial normalization is comparable to the MRI-based spatial normalization and therefore the use of individual MR images is not essential to obtain reproducible [^11^C]ABP688 quantification as we previously also confirmed for rats (29). This could be related to the fairly high spatial information in [^11^C]ABP688 PET images by the abundancy of mGluR5 throughout the brain. However, it is important to note that a disease condition might have a detrimental effect on the accuracy of the PET template for spatial normalization due to an altered signal. Therefore, we included diseased HD mice as in HD the levels of mGluR5 are known to be altered (30, 31). Importantly, imaging HD mice did not invalidate the high agreement between the 2 spatial normalization approaches.

### Test-retest stability of [^11^C]ABP688 PET imaging in mice

As reproducible quantification is fundamental in order to perform interventional and longitudinal studies, we examined the test-retest stability of [^11^C]ABP688 PET in healthy and diseased mice. Both BP_ND_ and V_T_ values showed a very low relative group difference between test and retest (< 5%) as well as negligible bias with Bland-Altman plots. Even though the average values between test and retest were highly reproducible, standard deviations of these measurements were relatively large, indicating high reproducibility on a group-level but limitations for the single animal. Nonetheless, these findings are in line with previous test-retest studies in rats and baboons, where a percentage difference below 10% was reported (11, 32, 18), with no statistically significant changes between the 2 scans. On the contrary, in a test-retest study in humans, a significant increase in BP_ND_ values was reported in the retest measurement, with regional differences up to 73% (15, 20). Recent studies confirmed test-retest regional increased uptake of [^11^C]ABP688 during retest scan in rhesus monkeys (19). This increased [^11^C]ABP688 binding during the retest scan could be related to a circadian variation of the mGluR5 during the day. In line with this hypothesis, a previous study comparing sleep deprived subjects to controls reported increased [^11^C]ABP688 binding following sleep deprivation (33). Additionally, a recent report showed that [^11^C]ABP688 binding in rats is significantly different during distinct phases of the day, supporting the hypothesis that glutamate binding to mGluR5 undergoes a circadian variation (34).

## Conclusion

In conclusion, we proposed a cardiac noninvasive input function (IDIF) to quantify the [^11^C]ABP688 volume of distribution in mice as well as the use of a cerebellar reference region and the SRTM method for the relative quantification of mGluR5 in mice.

Additionally, the good agreement between spatial normalization approaches indicates that a [^11^C]ABP688 PET template can be used for reproducible regional quantification during interventional or longitudinal studies in the mouse brain. Taken together, our results indicate that noninvasive relative quantification of [^11^C]ABP688 PET imaging can be perform in mice.

## Author contributions

JV, SSta, and LM conceived and designed the study. JV, DB, LK, DT, LW, AV, SStr, JW, CD, LM, and SSta were involved in execution of the experimental design, data acquisition and data interpretation for the study. DB, JV, and SSta were involved in drafting and editing the manuscript and figures. All authors approved the final manuscript and they are accountable for the content of the work.

### Conflict of interest statement

This work was funded by CHDI Foundation, Inc., a nonprofit biomedical research organization exclusively dedicated to developing therapeutics that will substantially improve the lives of HD-affected individuals.

## Supplementary material

Supplementary Figure 1 Comparison of [^11^C]ABP688 striatal quantification in WT and HET Q175 mice (*n* = 6 per genotype) using volume of distribution (V_T_) based on Logan plot with AV shunt (inv) **(A)** and IDIF **(B)** as well as binding potential (BP_ND_) using SRTM **(C)**. WT, wild type; HET, heterozygous; AV, arteriovenous; IDIF, image-derived input function; SRTM, simplified reference tissue model; *p, p*-value. Data are represented as mean ± standard deviation.

Supplementary Figure 2 Average SUV TACs for the test and retest scans of WT and HET Q175 mice (*n* = 5 per genotype). STR, striatum; CB, cerebellum; WT, wild type; HET, heterozygous. Data are represented as mean ± standard error mean.

Supplementary Table 1 Scan parameters for each of the performed studies. AV, arteriovenous; IDIF, image-derived input function; WT, wild type; HET, heterozygous.

Supplementary Table 2 Relationship between V_T_ and DVR-1 [^11^C]ABP688 quantification based on invasive (AV shunt) and noninvasive input function (IDIF). AV, arteriovenous; IDIF, image-derived input function; DVR, distribution volume ratio; *r*, Pearson's correlation; *r*^2^, coefficient of determination.

Supplementary Table 3 Correlations between DVR-1 and BP_ND_ for [^11^C]ABP688. AV, arteriovenous; IDIF, image-derived input function; DVR, distribution volume ratio; BP_ND_, binding potential; SRTM, simplified reference tissue model; *r*, Pearson's correlation; *r*^2^, coefficient of determination.

Supplementary Table 4 Representative power analysis to detect striatal difference in [^11^C]ABP688 quantification between WT and HET Q175 mice at 6 months of age.
