# Supplementary figures and images for: Noninvasive Relative Quantification of [11C]ABP688 PET Imaging in Mice Versus an Input Function Measured Over an Arteriovenous Shunt
Source: Front Neurol. 2018 Jun 29;9:516. doi: 10.3389/fneur.2018.00516 (PMC6036254; doi:10.3389/fneur.2018.00516)

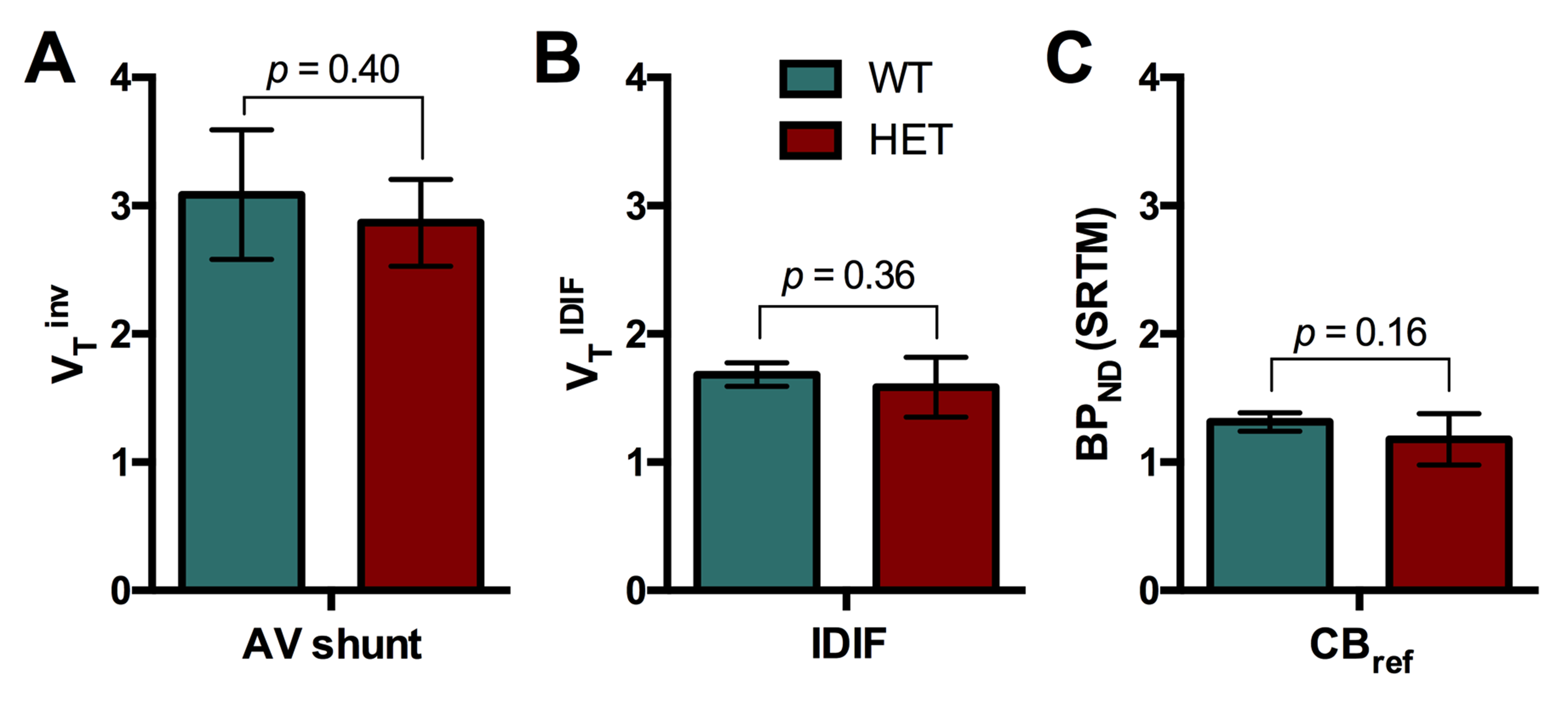

Supplement: Supplementary Figure 1 — Comparison of [11C]ABP688 striatal quantification in WT and HET Q175 mice (n = 6 per genotype) using volume of distribution (VT) based on Logan plot with AV shunt (inv) (A) and IDIF (B) as well as binding potential (BPND) using SRTM (C). WT, wild type; HET, heterozygous; AV, arteriovenous; IDIF, image-derived input function; SRTM, simplified reference tissue model; p, p-value. Data are represented as mean ± standard deviation. [file Image_1.TIFF]

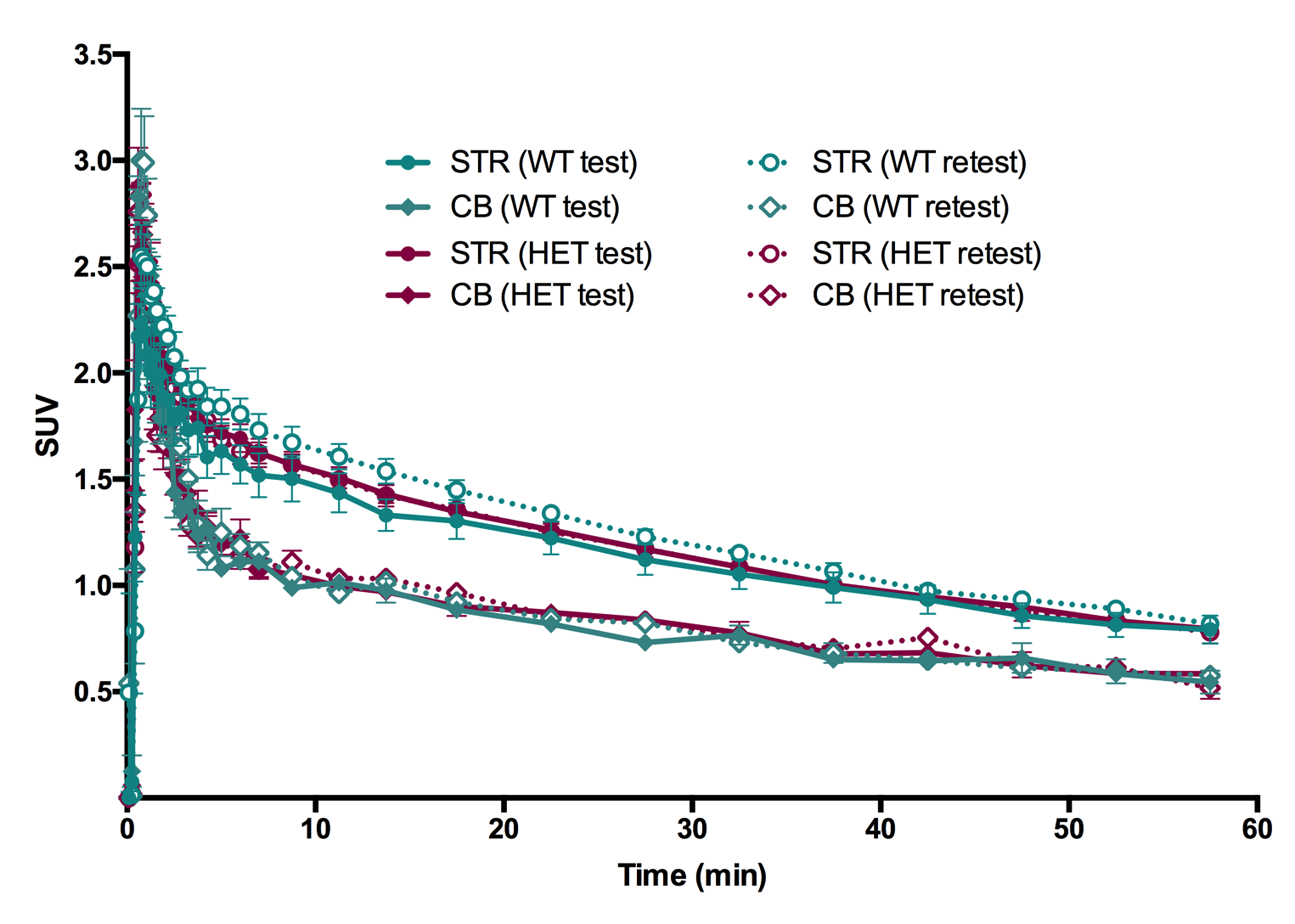

Supplement: Supplementary Figure 2 — Average SUV TACs for the test and retest scans of WT and HET Q175 mice (n = 5 per genotype). STR, striatum; CB, cerebellum; WT, wild type; HET, heterozygous. Data are represented as mean ± standard error mean. [file Image_2.TIFF]
