# Supplementary material for: Noninvasive Relative Quantification of [11C]ABP688 PET Imaging in Mice Versus an Input Function Measured Over an Arteriovenous Shunt
Source: Front Neurol. 2018 Jun 29;9:516. doi: 10.3389/fneur.2018.00516 (PMC6036254; doi:10.3389/fneur.2018.00516)
Supplement: Supplementary Table 1 — Scan parameters for each of the performed studies. AV, arteriovenous; IDIF, image-derived input function; WT, wild type; HET, heterozygous. [file Table_1.DOCX]

Supplementary Material

**Noninvasive relative quantification of [^11^C]ABP688 PET imaging in mice versus an input function measured over an arteriovenous shunt**

**Jeroen Verhaeghe^1^, Daniele Bertoglio^1^, Lauren Kosten^1^, David Thomae^1,2^, Marleen Verhoye^3^, Annemie Van Der Linden^3^, Leonie wyffels^1,2^, Sigrid Stroobants^1,2^, John Wityak^4^, Celia Dominguez^4^, Ladislav Mrzljak^4^, Steven Staelens^1^**

^1^Molecular Imaging Center Antwerp (MICA), University of Antwerp, Wilrijk, Belgium

^2^Department of Nuclear Medicine, Antwerp University Hospital, Edegem, Belgium

^3^ Bio-Imaging Lab, University of Antwerp, Wilrijk, Belgium

^4^CHDI Foundation, Princeton, NJ, United States of America

*** Correspondence:**

Prof. Steven Staelens

Molecular Imaging Center Antwerp (MICA)

Faculty of Medicine and Health Sciences

University of Antwerp

Universiteitsplein 1, Wilrijk, Belgium

Tel. +32 03265 2820

Email: [steven.staelens@uantwerpen.be](mailto:steven.staelens@uantwerpen.be)

# Supplementary Figures and Tables

**Supplementary Table 1 |** Scan parameters for each of the performed studies.

| **Study** | |  | **number of animals** | **body weight (g)** | **Injected radioactivity (MBq)** | **injected mass (μg/kg)** |
| --- | --- | --- | --- | --- | --- | --- |
|  |  |  |  |  |  |  |
| **AV shunt vs. IDIF** | | **WT** | 6 | 28.0 ± 1.3 | 6.20 ± 1.37 | 1.29 ± 0.12 |
|  |  | **HET** | 6 | 28.9 ± 1.2 | 5.99 ± 1.27 | 1.33 ± 0.04 |
| **Blocking** | **Baseline** | **WT** | 4 | 29.8 ± 2.3 | 3.93 ± 1.01 | 0.93 ± 0.04 |
|  | **MPEP** | **WT** | 4 | 34.9 ± 2.8 | 3.58 ± 0.61 | 0.68 ± 0.07 |
| **PET-template validation** | | **WT** | 15 | 30.1 ± 1.8 | 5.26 ± 1.30 | 1.31 ± 0.06 |
|  |  | **HET** | 16 | 28.7 ± 1.8 | 5.18 ± 1.08 | 1.27 ± 0.05 |
| **Test-retest** | **Test** | **WT** | 5 | 28.1 ± 2.1 | 5.06 ± 0.87 | 1.21 ± 0.09 |
|  |  | **HET** | 5 | 28.3 ± 1.8 | 5.43 ± 1.02 | 1.25 ± 0.08 |
|  | **Retest** | **WT** | 5 | 27.7 ± 2.0 | 4.60 ± 1.39 | 1.28 ± 0.05 |
|  |  | **HET** | 5 | 28.0 ± 1.4 | 5.19 ± 0.63 | 1.23 ± 0.04 |

AV = arteriovenous, IDIF = image-derived input function, WT = wild type, HET = heterozygous.
