# Supplementary material for: Noninvasive Relative Quantification of [11C]ABP688 PET Imaging in Mice Versus an Input Function Measured Over an Arteriovenous Shunt
Source: Front Neurol. 2018 Jun 29;9:516. doi: 10.3389/fneur.2018.00516 (PMC6036254; doi:10.3389/fneur.2018.00516)
Supplement: Supplementary Table 4 — Representative power analysis to detect striatal difference in [11C]ABP688 quantification between WT and HET Q175 mice at 6 months of age. [file Table_4.DOCX]

Supplementary Material

**Noninvasive relative quantification of [^11^C]ABP688 PET imaging in mice versus an input function measured over an arteriovenous shunt**

**Jeroen Verhaeghe^1^, Daniele Bertoglio^1^, Lauren Kosten^1^, David Thomae^1,2^, Marleen Verhoye^3^, Annemie Van Der Linden^3^, Leonie wyffels^1,2^, Sigrid Stroobants^1,2^, John Wityak^4^, Celia Dominguez^4^, Ladislav Mrzljak^4^, Steven Staelens^1^**

^1^Molecular Imaging Center Antwerp (MICA), University of Antwerp, Wilrijk, Belgium

^2^Department of Nuclear Medicine, Antwerp University Hospital, Edegem, Belgium

^3^ Bio-Imaging Lab, University of Antwerp, Wilrijk, Belgium

^4^CHDI Foundation, Princeton, NJ, United States of America

*** Correspondence:**

Prof. Steven Staelens

Molecular Imaging Center Antwerp (MICA)

Faculty of Medicine and Health Sciences

University of Antwerp

Universiteitsplein 1, Wilrijk, Belgium

Tel. +32 03265 2820

Email: [steven.staelens@uantwerpen.be](mailto:steven.staelens@uantwerpen.be)

# Supplementary Figures and Tables

**Supplementary Table 4 |** Representative power analysis to detect striatal difference in [^11^C]ABP688 quantification between WT and HET Q175 mice at 6 months of age.

Inv = invasive, IDIF = image-derived input function, BP_ND_ = binding potential, SRTM = simplified reference tissue model, SD = standard deviation, sem = standard error mean.
